# A website to identify shared genes in *Saccharomyces cerevisiae* homozygous deletion library screens

**DOI:** 10.1186/s12859-018-2212-4

**Published:** 2018-05-23

**Authors:** Mark D. Temple

**Affiliations:** School of Science and Health, Western Sydney University, Campbelltown Campus, Locked Bag 1797, Penrith South DC, NSW 1797 Australia

**Keywords:** Yeast, *Saccharomyces cerevisiae*, Database, Deletion library, Venn intersection

## Abstract

**Background:**

The homozygous yeast deletion library includes approximately 4800 diploid strains each containing one deleted non-essential gene. Hundreds of publications have arisen through experimentation using this genome-wide biological resource. As part of this work over 677 genesets have been collated from these experiments representing the phenotypic responses of the library to a diverse set of chemical and physical challenges.

**Description:**

A website called the *Saccharomyces cerevisiae* Homozygous Deletion Library Tools (*Sc*Ho DeLiTo-96) has been developed with the primary goal of browsing and identifying genes shared between these responsive phenotypes (available at yeastdb.org). Geneset comparisons have been performed for each phenotype against all others to identify common genes. Genesets and other curated information are stored in a relational database and a website interface allows users to query and browse the data in an intuitive way to reveal commonality between selected phenotypic responses. The most commonly occurring genes in all of the stored phenotypes are highly over-represented in the GO slim term “cellular ion homeostasis” indicating that genes shared between phenotypes may highlight a common cellular response. Additionally, user derived genesets can be uploaded and intersected against the stored data to reveal common responses which may otherwise have been obscure.

**Conclusion:**

These tools provide a simple method to perform niche enquiries between datasets derived from the yeast deletion library.

## Background

The deletion library of the budding yeast *Saccharomyces cerevisiae* is a collection of single gene knockout mutants, each of which contains a deletion in one of the 5800 or so known protein-coding sequences [1]. Phenotypic screening of this library under many different growth conditions or chemical treatments has been invaluable to determine the effect that individual gene deletion has on a cellular response [2]. A set of homozygous diploid strains representing nearly 4800 deletants of non-essential genes is an integral part of this collection and its usefulness is characterized by many publications reporting their response to diverse treatments [3–6]. This paper describes the *Saccharomyces cerevisiae* Homozygous Deletion Library Tools (*Sc*Ho DeLiTo-96) which is built upon a database of results taken from over 100 and 50 published papers that report deletion library screens using the 96-well plate method. The tools are available at http://yeastdb.org. These tools include a collection of dynamic and responsive webpages for the identification of common genes between these published phenotypic genesets. These tools can be used for data browsing to identify all genesets that are similar to one of the curated genesets. Users can also input their own geneset to identify if there are other similar responsive sets. Common genes between genesets are determined using a hypergeometric distribution statistical tests [7] without correction. There is a graduation in the degree of similarity between these genesets and whilst the ubiquitous *p*-value cut-off of 0.05 is an accepted criteria to identify commonality, further interpretation of the result is required since not all gene products are of equal functionality to the cell.

There are an abundance of website and databases that service the yeast research community with the *Saccharomyces* Genome Database (SGD) [8] being the most prominent. Indeed SGD have external links to over 40 other resources covering nucleic acid, genome and protein data, expression data, localization data, phenotype data, interactions data, literature and other general resources. Amongst these, *ScHo* DeLiTo-96 fills a niche to address browsing and analyses of datasets derived from the homozygous diploid deletion library. These tool pages provides links from ORF/gene names to SGD for further enquiry although for convenience some rudimentary annotation is provided directly. These tools are focused on quickly and easily identifying similarities between responsive genesets which may or may not have been expected based on the topic of research alone. Common genesets may be an indicator of a shared biological response and it is hoped that identifying these will be useful for data mining and for the analyses of new data.

## Construction and content

**Fig. 1:**
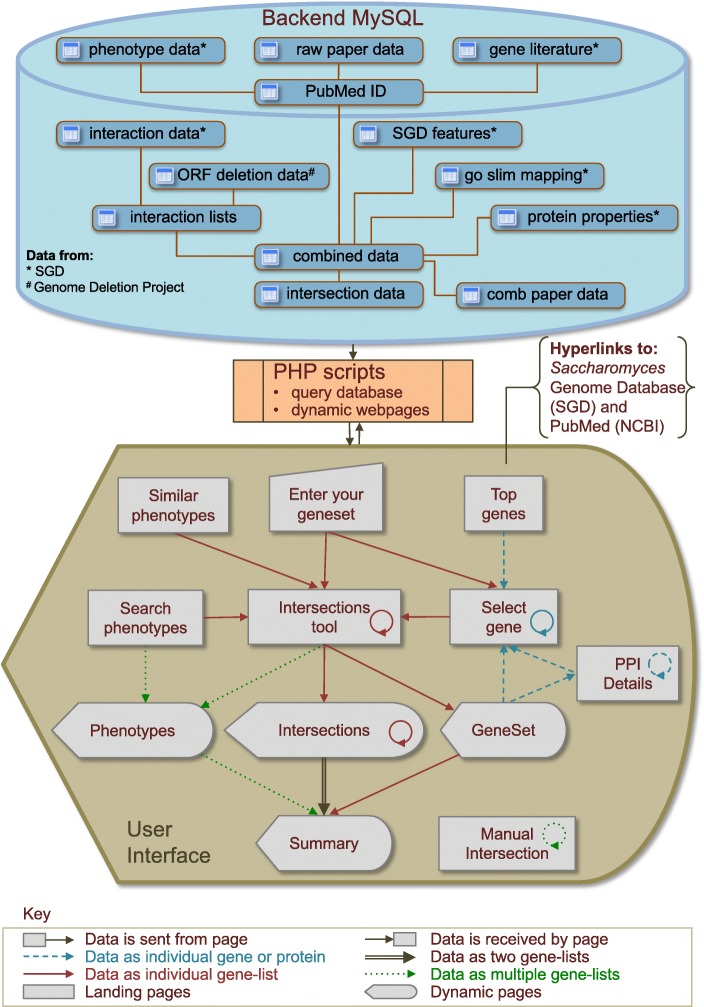
Workflow of the *ScHo* DeLiTo-96 pages. The database is compiled from various tab files curated by SGD and data curated from original publications. PHP scripts are used to pre-process these data into tables that are queried to produce the interactive webpages. Users can browse and select phenotypes or enter their own geneset through these webpages to begin intersection analyses towards identify common genes between phenotypes. Arrows indicate flow of data between webpage scripts, circular arrows indicate that data may by reloaded into the same page

A literature search was performed to identify publications that report screens of the *S. cerevisiae* homozygous diploid deletion library against various chemical or physical challenges using the 96-well plate method. It is thought that focusing on a single strain type and similar experimental approach will provide a reliable determination of similarity between experimental results. At the time of writing 167 papers were found and from these 677 distinct genesets (the responsive phenotypes) were derived. Many of these phenotypes were taken from prior curated papers in the SGD phenotype_data.tab and gene_literature.tab files and from these 106 and 4 relevant papers were obtained, respectively. Results from a further 57 papers were taken directly from the actual publications. These data were compiled in a MySQL database and an overview of the schema for this is shown in Fig. 1. These responsive phenotypes were added to the combined_data MySQL table and totalled 32,024 unique entries. In the first instance, each entry consisted of the systematic feature name, gene name, name of the publication and name of the responsive phenotype. Additionally the PubMed ID and SGD ID were stored to later establish hyperlinks to these external databases. Annotations of the deleted feature were added to this table which were taken from the SGD_features.tab, including the chromosome details, description and feature qualifier (e.g. whether the feature is a verified or dubious). Rudimentary Gene Ontology terms (process, component and function) were added from the SGD go_slim_mapping.tab file. Lastly, nearest neighbour protein-protein interactions data (genetic and physical) were added from the SGD interaction_data.tab file (derived from [9]). Links to these source data files are provided on the ‘Resources’ page. Furthermore, nearest neighbour interactions to genes that are essential or not available have been marked within the MySQL table since these are absent from the deletion library screen data, these are highlighted in the *ScHo* DeLiTo-96 tool pages and may be of interest to the user.

### Identification of common genesets

Further pre-processing of these genesets was performed to facilitate browsing of these data. Scripts written in PHP were used to intersect each phenotypic geneset against those from all other publications to identify common genes that are responsive to different treatments. These results were stored in the intersection_data table. Phenotypes from the same publication were not intersected against each other as these are often highly related (e.g. responses to a higher concentration of the same compound or to a related compound). Additionally it is assumed that relationships between these have already been characterised by the principal investigators. In total 1383 intersections (shared genesets) were identified from over 14,000 pairwise combinations of sets that had a *p*-value less than or equal to 1 × 10^− 12^. Note the ‘manual intersection’ page facilitates the intersection of any two papers of interest using a higher p-value. However, this highly stringent p-value was chosen since there is relatively a high degree of similarity between some reported genesets, for instance 34 deletants each occur in at least 50 of the 677 phenotypic sets. To emphasise this point, control data was compiled multiple times consisting of 100 genesets of randomly chosen genes of between 20 and 100 genes from the deletion library. Intersection of these control data typically revealed that no intersections passed this stringent filter.

Each shared geneset of the interaction_lists table was further annotated with common biological properties (stored in the combined_data table). In the first instance, common GO term mappings for process, function and component were pooled for each gene of the shared set and those over represented having a *p*-values of less that 0.05 were stored. Similarly, over-represented nearest neighbours common to each set were identified and stored for both physical and genetic protein-protein interaction data [9]. Lastly a global overview of these data were stored in the comb_paper_data table to summarise the numbers of intersections identified for each phenotype in the database. This pre-processed table is used as the backend for the homepage of the website which provides a summary page to launch the various browsing, intersection or annotation tools.

## Utility and discussion

**Fig. 2:**
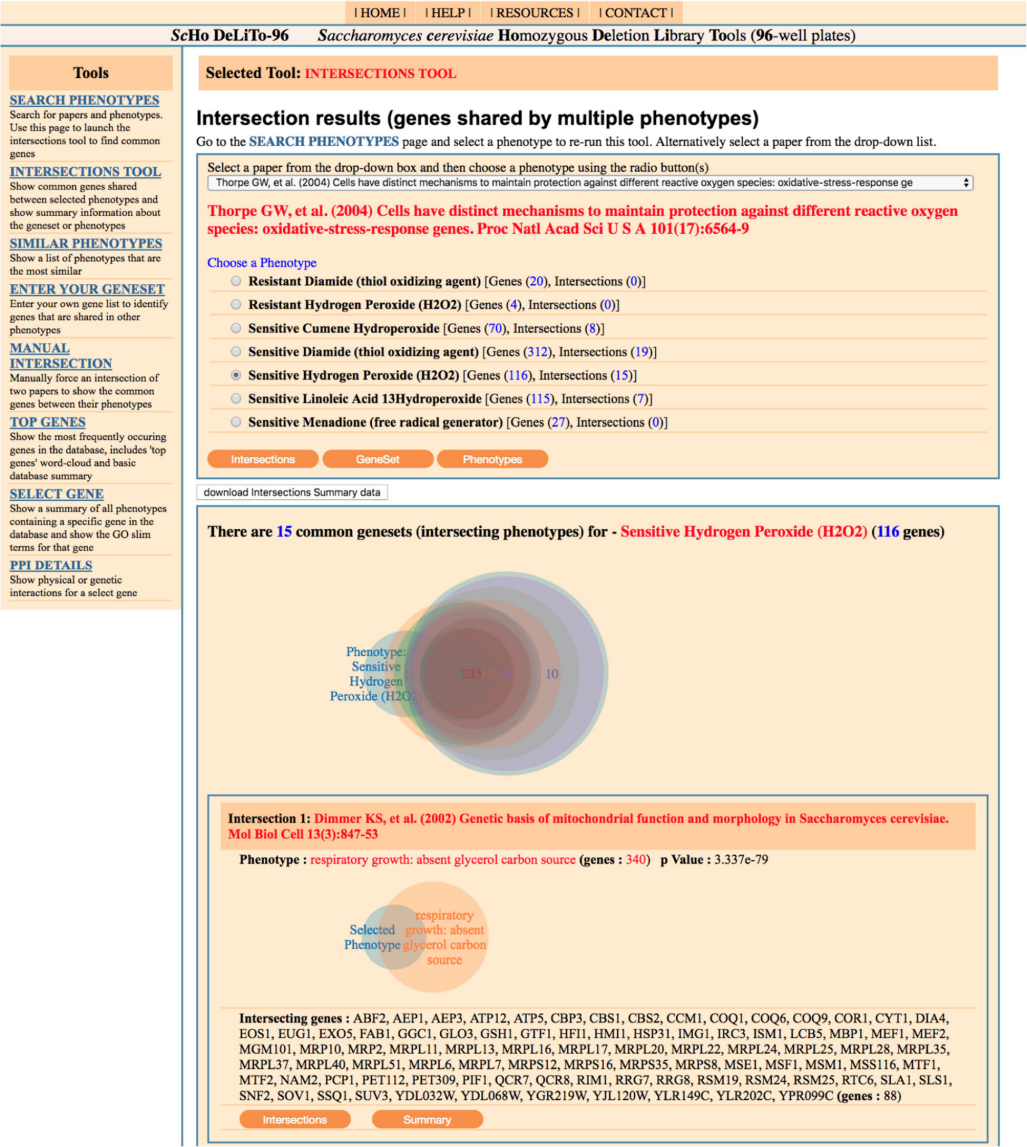
Screenshot of an enquiry from the ‘Intersections tool’ page. The selected paper reports 7 phenotypes and below these are three options (the ‘Intersections’, ‘GeneSet’ and ‘Phenotypes’ buttons) to further enquire about the selected phenotype. The page snippet shown is a result of the ‘Intersections’ option (which is shown by default). The selected ‘Sensitive Hydrogen Peroxide’ phenotype indicates that 15 other phenotypes in the database share common genes. Only the first of these is shown in the figure, having 88 common genes (from a total of 116 in the selected phenotype). Selection of the ‘GeneSet’ button provides further information itemised for each gene whereas the ‘Phenotypes’ button lists details shared across the phenotypes

Each section below refers to an individual page of the site and hyperlinks to these are available on the left side menu which is accessible throughout the *ScHo* DeLiTo-96 pages. This menu and the intersections tool page is shown in Fig. 2. All pages have the facility for users to download a tab separated text file to capture the results shown. Additionally a summary file of all of the phenotypic genesets and their annotations can be downloaded from the Resources page.

### Search phenotypes page

The ‘Search Phenotypes’ page is effectively the homepage of the site. This landing page provides a summary of all the research articles, phenotypes and intersections thereof that are stored in the database. At the time of writing the database contains 167 papers and from these 677 genesets have been compiled, each paper is hyperlinked to its occurrence in PubMed. The default behaviour of the page is to only show papers and their genesets if they have shared genes (intersections) with other papers, currently there at 111 papers containing 187 genesets that fit this description. There is a toggle button to show all of the papers in the database including those without shared genes. This page is designed so that users can easily browse genesets from a paper of interest to ascertain if it shares genes with another phenotype (and its associated publication). The list of papers is sorted alphabetically by first author. A search function is available on this page to find keywords in the titles, phenotypes, year and author fields. This filters the main table to show only those records that satisfy the search criteria. Listed below each paper title are the responsive deletant genesets reported by the paper, the number of genes contained in each phenotypic geneset and importantly the number of intersections with other phenotypic geneset. To the right of the publication title is a ‘Phenotypes’ link that launches a summary page listing all genes of the phenotypes, a summary of the Gene Ontology (slim terms) that are overrepresented in the phenotypes and a summary of protein-protein interactions that are highly connected to the phenotypes. Also, to the right of the publication title is an ‘Intersections’ link that shows the details of the shared genes identified (see the ‘Intersections tool’ page below) for each phenotypic geneset.

### Search phenotypes page

The ‘Intersections tool’ page shows the common genes shared between various phenotypes. This is determined by the intersection of a chosen phenotype (geneset) against all other genesets in the database (see example shown in Fig. 2). There are two methods to run the intersection scripts on this page. Firstly, phenotypic genesets can be piped across from other pages (such as the ‘Search phenotypes’ homepage) using various embedded ‘Intersections’ buttons (as shown in Fig. 1). Secondly, the phenotype of interest can be chosen and run from a dropdown list of papers at the top of the page itself. The page presents an intersection summary using a combined Venn diagram graphic [10] followed by a list of each intersection. This page also lists the name of the parent publication, the phenotype, the number of genes in each list, the identity of the shared genes and the associated *p*-value (determined by a hypergeometric distribution) used to filter out genesets whose similarity occurs by chance alone [7]. For each intersection a proportional Venn intersection diagram is generated that indicates the relative sizes of the ‘Selected Phenotype’ (geneset-A) against the found set (geneset-B) and the extent of intersection. Below each Venn diagram is a nested ‘Summary’ and another ‘Intersection’ button. The summary button provides the identity of genes common to the two phenotypes and a brief GO term summary of these (for a more detailed GO term analyses the use of a dedicated tool such as Gene Ontology Term Finder from SGD is recommended). The nested ‘Intersect’ button re-runs the intersection script with geneset-B as the selected phenotype.

Two additional sections are present on the ‘Intersections tool’ page, these are accessed via the ‘GeneSet’ and ‘Phenotypes’ buttons that link respectively to more information about the individual genes or about the selected phenotype as a whole. The ‘GeneSet’ button presents a graph indicating the location of genes from the selected phenotype on each of the 16 yeast chromosomes. Since yeast strains are ordered systematically in the 96-well plates according to their location on each chromosome, this graph may reveal any systematic error associated with a particulate plate or with adjacent wells of the plate. Below this is a summary table indicating how frequently each gene occurs in other phenotypes and the number of hits identified in various physical and genetic interaction networks. Lastly the page presents a more exhaustive table listing the interactions for each gene. Within these lists the availability of each gene in the deletant collection is indicated. This is important since some interacting partners in the protein-protein interaction data are not represented in the yeast deletion library as they may be essential (coloured red) or not available to be tested (coloured blue). This is because without these essential genes the particular strain of the deletion library cannot survive to be tested. Highlighting these may alert the user to a gene that is highly connected to the interacting set but which cannot be tested by the use of the homozygous knockout collection. Such ‘guilt by association’ would need to be tested by another method to see if it was involved in the phenotype. Each entry in the table also has buttons linking to the ‘Select gene’ and ‘PPI Details’ pages.

### Similar phenotypes page

This page highlights the most similar phenotypes in the database, that is those with the lowest *p*-values according to the hypergeometric distribution. For instance the most similar responsive phenotypes in the database are ‘chemical compound accumulation: decreased glycogen’ [11] and ‘respiratory growth: absent glycerol carbon source’ [12] that consist of 316 and 340 genes, respectively, and have 208 deleted genes in common. The page reports only the paper and phenotype names, however, the associated ‘Intersections’ button can be used to run further enquiry on the set of interest. Additionally the user can adjust the *p*-value to show more or less pairs of papers with common genesets.

### Enter your geneset page

This page allows users to enter their own geneset of interest and to intersect this against the database to identify genes that are common to another phenotype. The page accepts ORF names or gene names. The filtering p-value can be adjusted depending on the extent of similarity found and the extent of the users interest. A summary list of each query gene is also generated to verify that the geneset has been properly parsed by the scripts and additionally a link is provided from each ORF name to the comprehensive SGD database [8] in case further verification is required. This list is hidden by default and available by toggling the Show/Hide link. Additionally a ‘Select gene’ button is provided that links to the related ‘Select gene’ page (see below) to show all of the phenotypes in which the corresponding deletion strain occurs.

### Manual intersection page

This page gives the user the ability to select any two papers from the database and to manually intersect these against one another. Each paper can be selected from the two separate dropdown boxes. The *p*-value used to filter these results can be set arbitrarily to show shared genes that may otherwise be obscure. This allows the user to observe the shared genes and make an informed decision regarding their significance.

### Top genes page

This page shows the genes that occur most frequently in the database, i.e. the deletant strains that most frequently occur in various phenotypic responses. The extent of the list can be filtered by the minimum number of times a gene occurs in a phenotype. The default is set to show all genes that occur in 40 or more phenotypes. Interestingly, deletants with impaired vacuole fusion and vacuolar protein sorting occur frequently in this list indicating the important role these functions play in the response to many diverse treatments. Clearly these deletant strains are the most sensitive to a wide range of treatments and the biological function of these warrants further investigation in relation to this. Each gene is linked to the ‘Select gene’ page (see below). Additionally the ‘Top genes’ page reports the total number of publications, phenotypes, distinct genes and total gene entries in the database.

### Select gene page

Queries to this page are driven by the ‘Select gene’ button on other pages or by selecting a gene from the dropdown list at the top of the page. The results of this page show basic gene summary information and it lists all of the phenotypes in which the gene occurs. These phenotype details are initially shown but can be hidden by selecting the Show/Hide toggle. As part of the user workflow any phenotype in this list can be pushed directly to the ‘Intersections tool’ page for further enquiry using the ‘Intersections’ button.

### PPI details page

This page indicates the degree to which the selected gene/protein is connected within the protein-protein interaction data. These connected genes are tagged if they are known to be absent from the library. This tool is useful to highlight genes that are both highly connected to a gene of a phenotype and absent from the library. It maybe worthwhile testing these by another method to establish if they are involved in the phenotypic response. A gene/protein of interest can be piped in using the ‘PPI details’ button (from the GeneSet section of the ‘Intersection tool’ page) or selected directly from the dropdown list at the top of the ‘PPI details’ page itself. The tool generates a table entry for each physical or genetic interaction associated with the selected gene. Each protein/gene entry in the table contains an additional ‘PPI Details’ and ‘Select gene’ buttons to either re-run the ‘PPI details’ page centred on the newly selected gene or to run the previously described ‘Select gene’ page, respectively.

## Conclusions

The yeast deletion library represents a powerful resource for genome-wide identification of genes whose absence affects the cellular responses to a wide range of treatments. The *ScHo* DeLiTo-96 site reported here provides further means for the analyses of deletion library datasets through the identification of similarly responsive sets reported by other investigators. Whilst there are many other high quality sites for yeast focused data investigation, this site provided a means to search similar data types and avoids the complication of strain specific affectations since all members have the same genetic background. New datasets may be investigated prior to publication using this tool to identify relationships between phenotypes that may be otherwise obscure or difficult to identify. Additionally, researchers can browse and mine the data with a specific author or phenotypic response in mind. The database will be updated quarterly from source files and newly published screen data. Researchers in the field are encouraged to alert the author if they are aware of a publication that has been inadvertently omitted from the current iteration of the tool or if they have ideas to further expand the scope of these tools.
